# Committees and the Shortage of Resident Medical Officers : Is There Shortage?

**Published:** 1914-09-19

**Authors:** 


*To the Editor of* The Hospital.

### Committees and the Shortage of Resident Medical Officers: Is there Shortage?

*To the Editor of* The Hospital.

Sir,—It is possible that the shortage of resident medical officers which has afflicted the general hospitals for some time and which appears to be reaching a climax at the present moment may not be so real as some people think.

To take an example: At the *Dreadnought* Hospital, Greenwich, where there has always been an ample supply of men, the war has depleted the junior staff by several of its members, but there is, on the other hand, a positive influx of assistance offered to fill any vacancies that may arise. Not only have members of the honorary medical staff offered to act as house surgeons, but members of the teaching staff of the London School of Clinical Medicine have signified their willingness to act in a similar capacity. Several graduates in consulting practice have offered their services as physicians and surgeons to out-patients and in the special departments, and many general practitioners have offered to give a portion of their time to tending the sick in the hospital.

In consequence of the large number of members of the teaching staff who have been called away, and on account of the improbability of any students from the Royal Navy, the Indian Medical Service, and other Government departments, the London School of Clinical Medicine has been closed for the next session. This has set free a certain number of men ready to help. There are available a large number of qualified men willing to assist in the London hospitals, among these the long list of medical officers seeking employment in both the Navy and Army, many of whom, it is conceivable, will never be called up. While they are waiting they are ready enough to come on and do H.P. or H.S., and some of the senior ones to take duty in the out-patient consulting room.

On the whole the hospitals are not likely to suffer seriously by reason of the depletion of the senior and junior staff on account of the war.—I am, etc.,

September 14, 1914. A Hospital Officer.

